# Beneficial Effects of *Akkermansia muciniphila* Are Not Associated with Major Changes in the Circulating Endocannabinoidome but Linked to Higher Mono-Palmitoyl-Glycerol Levels as New PPARα Agonists

**DOI:** 10.3390/cells10010185

**Published:** 2021-01-19

**Authors:** Clara Depommier, Rosa Maria Vitale, Fabio Arturo Iannotti, Cristoforo Silvestri, Nicolas Flamand, Céline Druart, Amandine Everard, Rudy Pelicaen, Dominique Maiter, Jean-Paul Thissen, Audrey Loumaye, Michel P. Hermans, Nathalie M. Delzenne, Willem M. de Vos, Vincenzo Di Marzo, Patrice D. Cani

**Affiliations:** 1 Metabolism and Nutrition Research Group, Louvain Drug Research Institute, Walloon Excellence in Life sciences and BIOtechnology (WELBIO), UCLouvain, Université Catholique de Louvain, Av. E. Mounier, 73 B1.73.11, 1200 Brussels, Belgium; clara.depommier@uclouvain.be (C.D.); Celine.Druart@a-mansia.com (C.D.); amandine.everard@uclouvain.be (A.E.); rudy.pelicaen@uclouvain.be (R.P.); nathalie.delzenne@uclouvain.be (N.M.D.); 2 Endocannabinoid Research Group, Institute of Biomolecular Chemistry, Consiglio Nazionale delle Ricerche, 80078 Pozzuoli, Italy; rmvitale@icb.cnr.it (R.M.V.); fabio.iannotti@icb.cnr.it (F.A.I.); 3 Quebec Heart and Lung Institute Research Centre, Université Laval, Quebec City, QC G1V 0A6, Canada; cristoforo.silvestri@criucpq.ulaval.ca (C.S.); nicolas.flamand@criucpq.ulaval.ca (N.F.); 4 Centre NUTRISS, Institute of Nutrition and Functional Foods, Université Laval, Quebec City, QC G1V 0A6, Canada; 5 Pôle EDIN, Institut de Recherches Expérimentales et Cliniques, UCLouvain, Université catholique de Louvain, 1200 Brussels, Belgium; dominique.maiter@uclouvain.be (D.M.); jeanpaul.thissen@uclouvain.be (J.-P.T.); audrey.loumaye@uclouvain.be (A.L.); michel.hermans@uclouvain.be (M.P.H.); 6 Division of Endocrinology and Nutrition, Cliniques Universitaires St-Luc, 1200 Brussels, Belgium; 7 Laboratory of Microbiology, Wageningen University, 6708WE Wageningen, The Netherlands; willem.devos@wur.nl; 8 Human Microbiome Research Program, Faculty of Medicine, University of Helsinki, 00014 Helsinki, Finland

**Keywords:** endocannabinoids, endocannabinoidome, *Akkermansia muciniphila*, human, metabolic syndrome, obesity, mono-palmitoyl-glycerol, peroxisome proliferator-activated receptor alpha (PPARα)

## Abstract

*Akkermansia muciniphila* is considered as one of the next-generation beneficial bacteria in the context of obesity and associated metabolic disorders. Although a first proof-of-concept of its beneficial effects has been established in the context of metabolic syndrome in humans, mechanisms are not yet fully understood. This study aimed at deciphering whether the bacterium exerts its beneficial properties through the modulation of the endocannabinoidome (eCBome). Circulating levels of 25 endogenous endocannabinoid-related lipids were quantified by liquid chromatography with tandem mass spectrometry (LC-MS/MS) in the plasma of overweight or obese individuals before and after a 3 months intervention consisting of the daily ingestion of either alive or pasteurized *A. muciniphila*. Results from multivariate analyses suggested that the beneficial effects of *A. muciniphila* were not linked to an overall modification of the eCBome. However, subsequent univariate analysis showed that the decrease in 1-Palmitoyl-glycerol (1-PG) and 2-Palmitoyl-glycerol (2-PG), two eCBome lipids, observed in the placebo group was significantly counteracted by the alive bacterium, and to a lower extent by the pasteurized form. We also discovered that 1- and 2-PG are endogenous activators of peroxisome proliferator-activated receptor alpha (PPARα). We hypothesize that PPARα activation by mono-palmitoyl-glycerols may underlie part of the beneficial metabolic effects induced by *A. muciniphila* in human metabolic syndrome.

## 1. Introduction

Over the past two decades, the gut microbiota has been pinpointed as a key factor involved in the etiology of obesity-associated comorbidities (reviewed in [1,2,3]). Extensive pre-clinical research has led to the identification of the symbiont *Akkermansia muciniphila* as a potential new target to tackle metabolic disorders (reviewed in [4,5,6,7,8]). Nowadays, this bacterium is qualified as a next-generation beneficial microbe [9]. We have previously shown that administration of *A. muciniphila* reduced the development of diet-induced obesity and insulin resistance in mice, notably through reinforcement of the gut barrier function [10,11,12,13]. These results were later confirmed in several studies using different mouse models of obesity but were also largely extended to other metabolic disorders [14,15,16,17,18,19]. Our team has recently succeeded in the translational investigation by implementing the first human intervention consisting of daily ingestion of *A. muciniphila* for 3 months [20]. This study showed that administration of *A. muciniphila,* either alive or pasteurized, is safe and well-tolerated in adults suffering from prediabetes and metabolic syndrome (Microbes4U^®^ cohort) [20]. Beyond safety and feasibility assessments, this previous study demonstrated that the pasteurized form of *A. muciniphila* limits the worsening of cardiometabolic disorders associated with overweight and obesity [20]. Although many metabolic outcomes and potential causal factors were identified, some parts of the mechanism of action have yet to be uncovered. In this context, the endocannabinoid (eCB) system has emerged as an important endogenous signaling system to explore. Indeed, this complex system encompasses bioactive lipids and their receptors and is involved in a variety of physiological processes, including regulation of appetite, energy metabolism, and inflammation [21,22,23,24]. The arachidonic acid (AA) derived endocannabinoids, *N*-arachidonoyl-ethanolamine, also known as anandamide (AEA) and 2-arachidonoyl-glycerol (2-AG), are endogenous ligands of cannabinoid receptors 1 (CB_1_) and 2 (CB_2_) and have been for a long time the most widely studied members of their families of lipids, i.e., the long-chain *N-*acyl-ethanolamines (NAEs) and the monoacyl-glycerols (MAGs), respectively [25]. AEA and 2-AG share biosynthetic and degrading pathways with these congeners, which are often referred to as “eCB-like” mediators [26]. The NAEs include among others, *N*-palmitoyl-ethanolamine (PEA), *N*-stearoyl-ethanolamine (SEA), *N*-oleoyl-ethanolamine (OEA), *N*-linoleyl-ethanolamine (LEA), *N*-eicosapentanoyl-ethanolamine (EPEA), and *N*-docosahexanoyl-ethanolamine (DHEA), while MAGs include 2-oleoyl-glycerol (2-OG), 2-linoleoyl-glycerol (2-LG), 2-palmitoyl-glycerol (2-PG) among others. Together with their receptors, anabolic and catabolic enzymes, the eCBs and eCB-like mediators form the expanded eCB system, or endocannabinoidome (eCBome) [27]. Increasing evidence has suggested that obesity and related complications are associated with an altered eCBome both in mice models and in humans [13,23,28,29,30,31,32]. For instance, individuals with increased visceral fat mass exhibited increased levels of MAGs, while levels of some NAEs were shown to be higher in individuals with overall elevated adiposity [33]. Furthermore, obesity is characterized by CB_1_ upregulation in the liver and intestine [34,35,36,37]. In the latter tissue, CB_1_ over-activation may contribute to increased intestinal permeability and subsequent chronic inflammation, both being hallmarks of obesity-related complications [37,38]. Hence, CB_1_ agonists such as AEA are known to elicit gut permeability (reviewed in [28]). By contrast, pharmacological blockade of the CB_1_ receptor by an antagonist results in bodyweight reduction, decreased insulin resistance, and reduced endotoxemia in a model of diet-induced obesity [38]. In humans, treatment with the CB_1_ inverse agonist rimonabant potentiates the weight loss induced by a hypocaloric diet [39]. However, this drug was withdrawn from the market due to psychiatric side effects.

In view of their respective important influence on metabolism, various reports discussing the existence of a dialogue between the microbiota and the eCBome have been published [28,37,40]. Noteworthy, preventive effects of *A. muciniphila* on metabolic endotoxemia are associated with an attenuation of the CB_1_ upregulation observed in murine models of liver injury, colitis, and obesity [11,19,41]. Moreover, one study revealed that oral *A. muciniphila* administration increases the intestinal endogenous production of 2-PG, 2-OG, and 2-AG in obese mice [10]. These bioactive lipids are hypothesized to exert anti-inflammatory activities and positive effects on gut barrier integrity [28]. Altogether these data might be indicative of a possible modulation of the eCBome by *A. muciniphila*. In view of the aforementioned literature, we hypothesized that *A. muciniphila* might exert its beneficial metabolic effects by promoting the restoration of an altered eCBome in overweight and obese individuals newly diagnosed with metabolic syndrome and prediabetes. To test our hypothesis, the circulating levels of 25 endogenous eCBome-related lipid mediators were quantified in the plasma collected from the Microbes4U© study by liquid chromatography with tandem mass spectrometry (LC-MS/MS). We scaled the modification in the eCBome by using both univariate and multivariate methods.

The orphan G-protein coupled receptors (GPR) 18, 55, 110, 119, the transient receptor potential vanilloid type-1 (TRPV1) and 2- (TRPV2) channels, and the peroxisome proliferator-activated receptor (PPAR) α and γ are part of the non-classical receptors known to interact with certain lipids of the eCBome [26,42]. Of particular interest, activation of these latter nuclear receptors has been associated with positive outcomes in the context of metabolic syndrome [42,43]. PPARα is a key sensor of fatty acid flux, whose activation upregulates genes implicated in lipid metabolism (i.e., fatty acids transport, beta-oxidation), thus promoting uptake, use, and oxidation of free fatty acids [44,45]. Besides lipid metabolism, PPARα signaling also governs the expression of target genes involved in carbohydrate metabolism in the liver and skeletal muscle, while in the adipose tissue, the thermogenic program has been shown to be partially under the regulation of PPARα [43,46,47]. Importantly, activation of PPARα by OEA and PEA mediates anorexigenic, and anti-inflammatory effects, respectively [42,48], as well as mitigating altered intestinal membrane permeability [49]. On the other hand PPARγ activation by its agonists, which include AEA [50], has been associated with adipogenesis and enhancement of insulin sensitivity [51]. Because of these reasons, our experimental design included the screening, in silico and in a functional in vitro assay, on PPARα and γ, of any eCBome lipid mediator that we would have seen altered following treatment with *A. muciniphila*.

We report that the beneficial impact of *A. muciniphila* observed in the Microbes4U© cohort is independent of a major and generalized shift in eCBome mediators, but we identify a potential influence on the plasma levels of mono-palmitoyl-glycerols. We also report for the first time that 2-PG is an efficacious endogenous PPARα, but not PPARγ, agonist. These novel observations may have important clinical implications in the context of preventive and therapeutic approaches using *A. muciniphila* as an adjuvant.

## 2. Materials and Methods

### 2.1. Participant and Study Design

The biological samples used in the present study originated from the Microbes4U© cohort. Individuals were recruited at the Cliniques Universitaires Saint-Luc in Brussels between 2015 and 2018. The cohort consists of 32 overweight or obese subjects (body mass index > 25 kg/m^2^) newly-diagnosed with a metabolic syndrome and with a prediabetes state as well as an insulin sensitivity < 75% [52,53]. Metabolic syndrome was diagnosed according to the National Cholesterol Education Program Adult Treatment Panel III definition, that is, at least three of the five following criteria: fasting glycemia > 100 mg/dL; blood pressure ≥ 130/85 mmHg or antihypertensive treatment; fasting triglyceridemia ≥ 150 mg/dL; high-density lipoprotein (HDL) cholesterol < 40 mg/dL for men, <50 mg/dL for women; and/or waist circumference > 102 cm for men, >88 cm for women. The subjects were naïve for medications influencing the parameters of interest (glucose-lowering drugs such as metformin, DPP4 inhibitors, GLP-1 receptor agonists, acarbose, sulfonylureas, glinides, thiazolidinediones, sodium-glucose cotransporter-2 inhibitors, insulin, lactulose, consumption of antibiotics in the previous 2 months before the inclusion, glucocorticoids, immunosuppressive agents, statins, fibrates, orlistat, cholestyramine, or ezetimibe). Other exclusion criteria encompassed baseline HbA1c > 7.5%, presence of acute or chronic progressive or chronic unstabilized diseases; inflammatory bowel disease or irritable bowel syndrome; diabetic gastrointestinal autonomic neuropathy (such as gastroparesis or reduced gastrointestinal motility); previous bariatric surgery; any surgery in the 3 months before the study or planned for 6 months after enrolling; regular physical activity (>30 min of sports activities 3 times a week); alcohol consumption (>2 glasses per day); consumption of dietary supplements (omega-3 fatty acids, probiotics, prebiotics, plant stanols/sterols) in the month before the study; consumption of more than 30 g of dietary per day; consumption of vegetarian or unusual diets; lactose intolerance or milk protein allergy; gluten intolerance; pregnancy or pregnancy planned in the 6 months after enrolling.

Following inclusion, individuals were randomly assigned to ingest daily for 3 months either 10^10^ cells of alive *A. muciniphila*, 10^10^ cells of pasteurized *A. muciniphila* in PBS containing glycerol, or a placebo (consisting of an equivalent volume of sterile PBS containing glycerol). Pasteurization consisted of heat treatment at 70 °C for 30 min of fresh *A. muciniphila*. Participants and physicians were both blinded to treatment allocation. At baseline and the end of the intervention, participants were asked to come to the hospital in a fasting state for blood sampling. Fasting state was required to reduce confounding factors related to nutrient intake and therefore to limit variability across individuals in the measurement of eCBs and their congeners. Written informed consent was obtained from each participant and the study protocol was approved by the Commission d’Ethique Biomédicale Hospitalo-facultaire of the Université catholique de Louvain. The study was registered at https://clinicaltrials.gov as trial no. NCT02637115.

### 2.2. Biochemical Analysis

Plasma samples were collected after overnight fasting (8 h minimum) in lithium-heparin-coated tubes, were brought rapidly to the research laboratory, and kept on ice. Plasma was immediately isolated from whole blood by centrifugation at 4200× *g* for 10 min at 4 °C and stored at −80 °C for further analyses.

The MAGs and NAEs extraction from plasma was done as described previously [54]. In brief, samples were mixed with TRIS (pH 7.4, 50 mM) to a final volume of 500 µL. Then, we added toluene (2 mL) containing the ISTD to the samples. The latter were vortexed for 1 min, centrifuged at 4000× *g* for 5 min without brakes, and put in an ethanol-dry-ice bath (−80 °C) to freeze the aqueous phase (bottom). We collected the organic phase (top) which was evaporated to dryness under a stream of nitrogen. Samples were reconstituted in 25 μL of HPLC solvent A (water with 0.05% acetic acid and 1 mM NH4+) and 25 μL of solvent B (acetonitrile/water, 95/5, v/v, with 0.05% acetic acid and 1 mM NH4+). A 40 μL aliquot was injected onto an RP-HPLC column (Kinetex C8, 150 × 2.1 mm, 2.6 μm, Phenomenex, Torrance, CA, USA). Quantification was achieved using a Shimadzu 8050 triple quadrupole mass spectrometer (Shimadzu, Kyoto, Japan) using the same LC program as previously described [13]. For the MAGs containing unsaturated fatty acids, the data are presented as 2-MAGs although it represents the combination of 1(3)- and 2- isomers given the recognized acyl migration from the sn-2 to the sn-1 or sn-3 position. All samples were processed the same day and immediately analyzed. Each sample was processed and analyzed once as the analysis utilized all the available plasma. Samples were processed and analyzed by the LC-MS/MS operator in a blinded, randomized manner.

Abbreviations used for the eCBs mediators present in this paper are presented in Table 1.

*A. muciniphila* was quantified with quantitative PCR as previously described in Everard et al. [10]. Each assay was performed in duplicate in the same run. The cycle threshold of each sample was then compared with a standard curve (performed in triplicate) made by diluting genomic DNA (fivefold serial dilution) (DSMZ).

### 2.3. Computational Methods

Starting ligand geometry was built with Ghemical (version 2.99.2) [55], followed by initial energy minimization (EM) at the molecular mechanics level, using Tripos v5.2 force field parametrization [56], and then at the AM1 semi-empirical level. The molecule was then fully optimized using the GAMESS v2018_R3 program [57] at the Hartree–Fock level with the STO-3G basis set and subjected to HF/6-31G*/STO-3G single-point calculations to derive the partial atomic charges using the RESP procedure [58]. Docking studies were performed with AutoDock (version 4.2) [59], by using PPARα and PPARγ crystallographic structures (Protein Data Bank (PDB): 2P54 and 2F4B, respectively). Both proteins and ligands were processed with AutoDock Tools (ADT) package version 1.5.6rc1 [59] to merge non-polar hydrogens and calculate Gasteiger charges. Grids for docking evaluation with a spacing of 0.375 Å and 60 × 60 × 60 points, centered on the ligand-binding site, were generated using the program AutoGrid (version 4.2) included in Autodock (version 4.2) distribution. 100 molecular docking runs for each docking calculation were performed adopting a Lamarckian Genetic Algorithm (LGA) and the protocol already published [60]. Flexibility was used for all rotatable bonds of the docked ligands. For each docking run, the most populated cluster endowed with the best binding energy values were selected as representatives and underwent energy minimization with Amber package (version 16) [61] using ff14SB version of AMBER ff14SB force field for the protein and gaff parameters for the ligand. To perform MD simulations in solvent, the complexes were confined in TIP3P water periodic truncated octahedron boxes exhibiting a minimum distance between solute atoms and box surfaces of 10 Å, using the leap module of the AmberTools16 package (version 16). MD simulations were performed using a protocol published elsewhere [60]. The cpptraj module of AmberTools16 and program UCSF Chimera v1.10.1 [62] were used to perform MD analysis and to draw the figures, respectively.

### 2.4. Cell Culture, Transfection and Luciferase Assay

Fibroblast-like COS cells (ATCC CRL-1651) were propagated in a growth medium (GM) composed of Dulbecco’s modified Eagle’s medium (DMEM cat. n. 41966029; Thermo Fisher, Monza, Italy) supplemented with 10% fetal bovine serum (cat. n. 16000044; Thermo Fisher, Monza, Italy) and 1% Pen/Strep (cat. n. 15140122 Thermo Fisher, Monza, Italy) under standard conditions. After plating (in 24 well plate density; 5 × 10^4^ cells/well), the cells were transfected on the next day with the following plasmids: (a) pM1-hPPARα-Gal4 or pM1-hPPARγ-Gal4; (b) TK-MH100 × 4-Luc containing the UAS enhancer elements and; (c) *Renilla* luciferase (pRL, Cat. E2231; Promega, Milan, Italy) using lipofectamine 2000 (cat. n. 11668027; Life Technologies; Milan, Italy) One day before transfection, COS cells were plated at confluency of 0.5–2 × 10^5^ cells in 500 μL of growth medium without. The day next, when the cells reached about 80% confluence were transfected by adding directly into the media the transfection mix composed of 250 ng M1-hPPARα-Gal4 or pM1-hPPARγ-Gal4, 250 mg of TK-MH100 and 250 mg of Renilla mixed in 50 μL of Opti-MEM containing 2 uL Lipofectamine 2000 (DNA/Lipo mix used per well).The next day, the growth media was replaced with fresh media containing vehicle or compounds of interest (2-PG, Fenofibrate, Cat. No. 4113/50 or Rosiglitazone Cat. No. 5325/10, purchased from TOCRIS, Abingdon, UK). Dimethyl sulfoxide (DMSO) was used as the vehicle. On day 3, the cells were harvested and processed for analysis of luciferase activity using a GloMax Luminometer instrument (Promega, Milan, Italy) and the Dual-Luciferase Reporter Assay kit (cat. n. E1910 Promega, Milan, Italy) following published procedures [63].

### 2.5. Statistical Analysis

The fold change plot was realized in Rstudio (version 1.2.1335) using the “ggplot2” package (version 3.3.2). The mean fold change for each eCB congener was expressed as a percentage, according to the following formula:

Fold Change (%) = Mean ((T3 value − T0 value)/T0 value)_ind_

Where ‘ind’ referred to each participant.

The mean difference was then calculated for each eCB congener, per group, by subtracting the value obtained at T0 from the value obtained at T3 months for each participant, according to the following formula:

Mean difference = Mean_Group_ (T3 value − T0 value)_ind_

Kruskal-Wallis test was then used to compare mean differences between groups for each eCB congeners and the corresponding *p*-values were indicated with the fold changes.

The focused principal component analysis (fpca) centralized on *A. muciniphila* was realized using the “psy” package (version 1.1) on Rstudio (version 1.2.1335). The fpca visualized graphically and faithfully the Pearson correlation between the variable abundance in *A. muciniphila* and the eCB-related mediators, and between eCB-related mediators themselves. For the latter, the fpca can be interpreted as a PCA [64]. The red circle represents the cut-off value of 0.05 for the Pearson’s correlation *p*-value. For multivariate analysis, data were normalized by auto-scaling (standardization). Normalization, Principal Components Analysis (PCA), Partial Least Square-Discriminant Analysis (PLS-DA), and time-series heat map clustering analysis were carried out using MetaboAnalyst v4.0 online platform (https://www.metaboanalyst.ca/ accessed on 26 November 2020) [65,66]. Hierarchical clustering was performed using the Ward algorithm and separated by Euclidean distance with factors arranged visually by time point and by groups. We applied PCA to obtain an overview of the eCBome profile quantified at the end of the intervention, according to groups. We then performed a supervised PLS-DA to assess whether we could cluster groups according to their respective eCBome profile. Since PLS-DA is highly susceptible to overfit data, we validated the model with cross-validation using the parameters of multiple correlation coefficient (R2) and the cross-validated R2 (Q2). The method employed was the leave-one-out (LOO) cross-validation. In the validation step, an additional permutation test was also generated using 1000 cycles to evaluate the statistical significance of the separation.

For univariate analysis, the normal distribution of eCB-related mediators, either expressed as raw data or as the difference between the two main times points (i.e., T0 and T3 months), was tested using the Shapiro–Wilk test. We also took into account the appearance of box plots and Quantile–Quantile plots. For 1-PG, 2-PG, and their combination, the intervention effect was calculated by subtracting the value obtained at T0 from the value obtained at T3 months for each participant within each group according to the above formula. We named the delta obtained “mean difference”. We then calculated the “mean difference from the placebo group”, expressed as raw data and as a percentage, for both treatment groups by subtracting the “mean difference” of the active group to that of the placebo, according to the following formulas:

*Mean difference from placebo* = Mean _Group_ [(T3 value − T0 value)_ind_ − Mean difference _placebo_]

*Mean difference form placebo (%)* = Mean _Group_ [((T3 value − T0 value)/T0 value)_ind_ − Fold Change _placebo_]

Non-parametric two-tailed Mann–Whitney U-tests were used to assess significant differences between the mean differences of the two treated groups versus the mean differences of the placebo group. Non-parametric, matched-pairs Wilcoxon signed-rank, two-tailed, tests were performed to identify the differences between T0 and T3 within each group. Finally, Kruskal–Wallis tests, followed by post-hoc Dunn’s multiple comparisons test, were used to compare the “mean difference” across the three groups. All the aforementioned analyses and the corresponding graph were generated using the Prism software v.8.4.2 (GraphPad Software).

## 3. Results

### 3.1. Preliminary Time-Series Analyses

**Figure 1:**
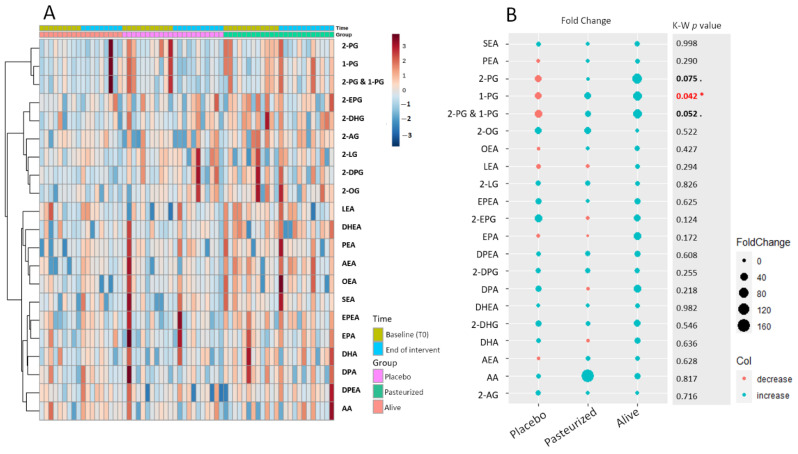
(**A**) Time-series hierarchical cluster analysis. The heat map visualized the levels of eCBome-related mediators according to each time and for each participant. Colored boxes indicate the normalized concentration of the corresponding eCB-related mediator from blue (lowest) to red (highest). Columns correspond to samples and rows to the eCB-related mediators. The algorithm for heatmap clustering was based on the Euclidean distance measure for similarity and Ward clustering algorithm. (**B**) Fold change in eCB-related mediators according to the group expressed in percentage. The size of the dot is proportional to the value of the fold change calculated within each group. The blue dots represent positive fold changes, meaning that the corresponding parameter had increased in average percentage following the treatment according to the baseline value, while the red dots represent negative fold changes, meaning that the corresponding parameter had decreased in average percentage following the treatment. The “K-W *p*-value” column shows the approximate *p*-value of the Kruskal–Wallis statistical test applied to the row delta taking into account the 3 groups. * Red writing: *p* < 0.05; bold writing 0.1 > *p*-value > 0.05. Placebo group, *n* = 11; pasteurized bacteria group, *n* = 12; alive bacteria group, *n* = 9. Abbreviations: see Table 1.

To begin our exploration of the dynamic changes that occurred at the level of the eCBome following the intervention, we first took a look at the individual variation for each eCB-related entity through the construction of a time-series hierarchical clustering (Figure 1A).

This approach allows us to obtain a global visualization of the normalized concentration of each eCB-related mediator for each pair of samples (i.e., before and after intervention in each individual). To facilitate the detection of potentially relevant variations within the eCBome the heat map was organized by timings and by groups. The corresponding matrix did not reveal any particular pattern indicating either no overall or more specific changes in the quantified eCBome after the treatment period or according to the groups. To help identify potential discrete modifications within the eCBome, we pursued our analyses by calculating the fold change, which is the mean differential value between the two main time points (i.e., T0 and T3) expressed in percentage for each eCB-related congener. We then plotted the resulting fold changes according to groups (Figure 1B). Interestingly, we observed that all eCB-related mediators increased in percentage average in the alive *A. muciniphila* group, while opposite trends were observed for some eCBs in the other two groups. We then assessed whether the row mean difference measured in the three groups were statistically different by statistical analysis. As the distribution of the eCB-related congeners followed a non-Gaussian distribution curve, we performed a non-parametric Kruskal–Wallis test. The resulting *p*-values are indicated in the left column of Figure 1A. Except for 1-PG, 2-PG, and the combination of both isomers, all *p*-values were above 0.1. To complete our time-series analyses, we then performed an unsupervised PCA, followed by the examination of the first three principal components (PCs). The resulting three-dimensional score plot failed to clearly separate groups based on the time of the study (Supplementary Figure S1A–D). Overall, these preliminary analyses suggested that the intervention influenced more specific entities of the eCBome, rather than the whole system. The next sections will be dedicated to the verification of this first conclusive statement.

### 3.2. Deeper Characterisation of the Effect of A. muciniphila on 1-PG and 2-PG Circulating Levels

**Figure 2:**
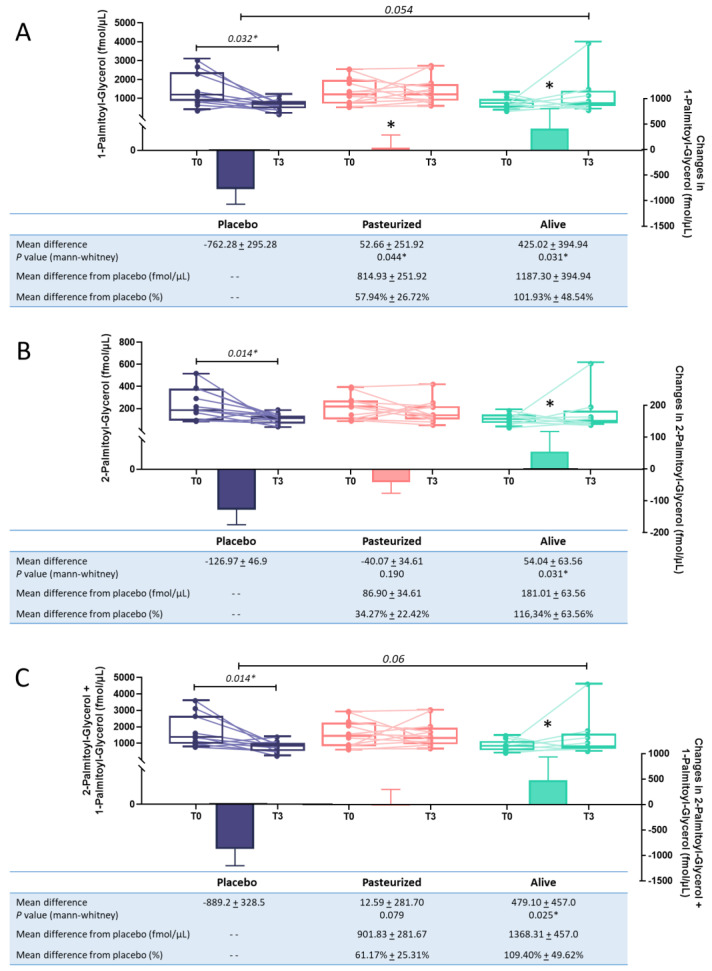
Evolution of plasmatic levels for 1-Palmitoyl-glycerol (**A**), 2-Palmitoyl-glycerol (**B**), and the combination of the 2 isomeric forms (**C**) following the intervention. Purple color was used for the placebo group, pink for the pasteurized group and green for the alive group. Differential values (mean difference and mean difference from placebo) are expressed as the mean ± s.e.m., either as raw data or as percentages. The “- -” in the table refers to “non applicable”. The bars represent the delta per group with the corresponding s.e.m., which corresponds to the mean difference between the value at the end of the intervention and the baseline value. Two-tailed Mann–Whitney U-tests were performed to compare the differential values of both treated groups versus the placebo group (intergroup changes). Below each plot are indicated the respective *p*-values and when the test is significant, the bars are marked with an asterisk. The horizontal lines represent the evolution of the raw values before and after the intervention. The box-and-whiskers plot illustrates the distribution of the raw values for each timing within each group. The line in the middle of the box is plotted at the median, while the superior and inferior limits of the box correspond to the 75th and the 25th percentiles, respectively. The whiskers correspond to the maximum and minimum values. Two-tailed matched-pairs Wilcoxon’s signed-rank tests were performed to verify changes from baseline (intragroup changes). When the difference is significant, a capped line is marked above the relevant group with the corresponding *p*-value. Changes between 0 and 3 months across the 3 groups were analyzed with Kruskal–Wallis test; group-wise comparisons were performed using Dunnet’s corrections for multiple testing. When the difference is close to significant, a line is marked above the relevant groups with the corresponding *p*-value. Placebo group, *n* = 11; pasteurized bacteria group, *n* = 12; alive bacteria group, *n* = 9. * *p* < 0.05.

During our previous analysis, we measured the fold change for each eCB, per group. The value obtained corresponds to the difference between the raw value after the intervention and the raw value at baseline, expressed in the percentage of average change. When comparing the average mean difference between groups for each eCB congeners, the corresponding statistical test identified a significant difference for 1-PG, and differences close to the significance threshold for 2-PG and the combination of the two isomers. Our next analysis aimed to further characterize these effects using univariate approaches. First, we compared the initial concentration measured for these compounds to the one measured at the end of the intervention, within each group. After 3 months, the placebo group exhibited significant reductions in circulating levels of 1-PG, 2-PG, and the combination of the two isomers (*p* < 0.05, T3 versus T0; Figure 2A–C).

When compared to the baseline values, no significant variations were measured in subjects treated with the pasteurized and the alive form of the bacterium. We then compared the differential values measured in the two treatment groups to that of the placebo group. Interestingly supplementation with both forms of the bacterium (i.e., alive or pasteurized) significantly increased the levels of 1-PG when compared to the placebo group. More specifically, the supplementation significantly increased by 57.94% and 101.93% the circulating levels of 1-PG in the pasteurized and the alive *A. muciniphila* group, respectively (*p* = 0.041; *p* = 0.031; Figure 2A). 2-PG levels were significantly increased by about 116% in the alive *A. muciniphila* group compared to the T3 levels observed in the placebo group (*p* = 0.031; Figure 2B). Conversely, this isomer level was not affected by supplementation with the pasteurized form of *A. muciniphila* (Figure 2B). Finally, a similar observation was made for the combination of both isomers, with a significant increase by 109% of the circulating levels in the alive *A. muciniphila* group when compared to the placebo differential value (*p* = 0.025). As we previously demonstrated that both supplementations were associated with a significant increase of the abundance of *A. muciniphila* recovered in the feces of the participants, with a more pronounced effect for the alive form [20], we performed focalized principal component analyses to investigate whether the fecal abundance of *A. muciniphila* was correlated to one or more eCB-related congeners. The analysis was performed for both timings and showed that none of the quantified eCBome mediators correlated significantly with the abundance of *A. muciniphila* (Supplementary Figure S2A–B).

### 3.3. Global eCBome Profiling Comparisons between Groups and Timing through Multivariate Analyses

**Figure 3:**
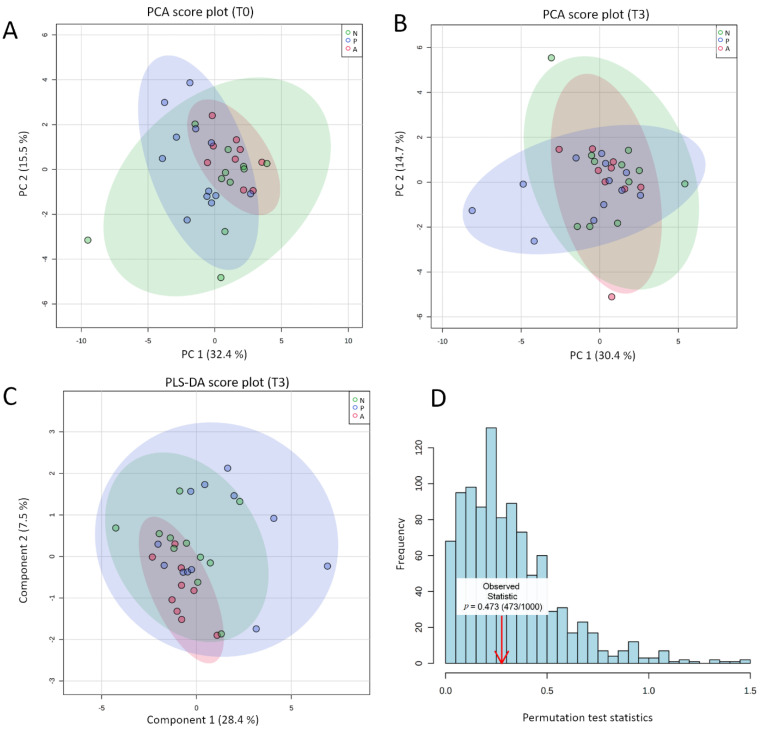
(**A**,**B**) 2D PCA Score plot for comparison of the global eCBome profile at baseline (**A**) and following the intervention (**B**) according to the group. (**C**) 2D PLS-DA cross-validated score plot for comparison of the global eCBome profile between groups following the intervention (time 3 months). The semi-transparent area is the 95% confidence region for each group. Variance explanation (%) for each PC/component is indicated. Color code: green ellipses correspond to the placebo group; blue ellipses correspond to the pasteurized group; red ellipses correspond to the alive group, with each dot representing one participant. (**D**) Result of the permutation test with separation distance (B/W) select for a statistical test, set permutation numbers: 1000. Analyses and graphs were performed and generated using MetaboAnalyst v4.0 (26 November 2020). Placebo group, *n* = 11; pasteurized bacteria group, *n* = 12; alive bacteria group, *n* = 9. Abbreviations: A, alive; N, non-treated (Placebo); P, pasteurized; PCA, principal component analysis; PC, principal component; PLS-DA; partial least square discriminant analysis; T0, time 0 month; T3, time 3 months.

After studying both individual and group-related variations by approximating both the raw values before and after the intervention and the corresponding fold changes, we investigated whether it was possible to cluster groups according to their respective eCBome mediator profiles determined at the end of the intervention. Beforehand, we checked the absence of initial bias due to the pre-existence of a baseline separation between groups. For this, we performed a PCA on the quantified baseline eCBome. The resulting two-dimensional PCA score plot showed that the groups were not different on the eve of the intervention (Figure 3A).

The same unsupervised analysis was performed on the eCBome quantified at the term of the study to assess whether the intervention resulted in a natural shift in the eCBome. The corresponding PCA score plot is shown in Figure 3B. The first two PCs explained 45.1% of the overall variance; 30.4% and 14.7% for PC1 and PC2, respectively. We observed that the three ellipses were superposed indicating that no natural separation existed between the three groups according to the eCBome (Figure 3B). Given the modest proportion of variance explained with the first two PCs, we generated score plots taking into account up to 4 principal components. However, none of the new 2-dimensional combinations resulted in a separation between groups (Supplementary Figure S3A). We next attempted to further explore whether it was possible to discriminate groups based on eCBome mediators using a supervised approach, the PLS-DA. PLS-DA is a dimension reduction method classically used in metabolomic studies for classification purposes. Provided that the model is validated, this approach allows for the identification of metabolites involved in group separation.

Like PCA, PLS-DA was performed on the eCBome quantified in the blood of participants at the end of the intervention. When the PLS-DA is performed and the resulting score plot indicates the separation between groups, the following cross-validation step is mandatory to certify that separation is statistically significant and not the result of random noise [67]. Similarly to PCA, the two-dimensional PLS-DA score plot exhibited superposed ellipses between groups (Figure 3C). Here also, exploration of the different possible combinations between components did not highlight separated clustering (Supplementary Figure S3B). The PLS-DA model validation was assessed through permutation tests based on the separation distance. The permutation using 1000 random tests showed a *p*-value of 0.473 (Figure 3D). These results aligned with the appearance of the score plot, for the certified absence of distinct clustering according to the eCBome profile. Moreover, regardless of the number of components selected, the value of the cross-validated R2 (Q2) stayed negative (Table 2).

However a good predictive model needs to have a high Q2 value, therefore, this indicated a poor predictive model, in line with our previous statement [67]. The absence of clear clustering added to the fact that the model could not be checked through cross-validation made it irrelevant to try to identify the eCB mediators that most influence the clustering of groups at time 3 months. To finally verify the absence of separation between timing, we performed PCA and PLS-DA within each group (Supplementary Figure S4A–F). Although for the alive *A. muciniphila* group, the PLS-DA score plot suggested a slight clustering between the two timings (Supplementary Figure S4F), both the permutation test and the LOO-cross validation pointed to the invalidity of the model (R2: 0.86, Q2: 0.20 for the first 4 components). Accordingly, no eCB-related mediator was identified as relevant to discriminate the two timings.

### 3.4. In Silico Identification and In Vitro Validation of 2-PG as an Endogenous Activator of PPARα

**Figure 4:**
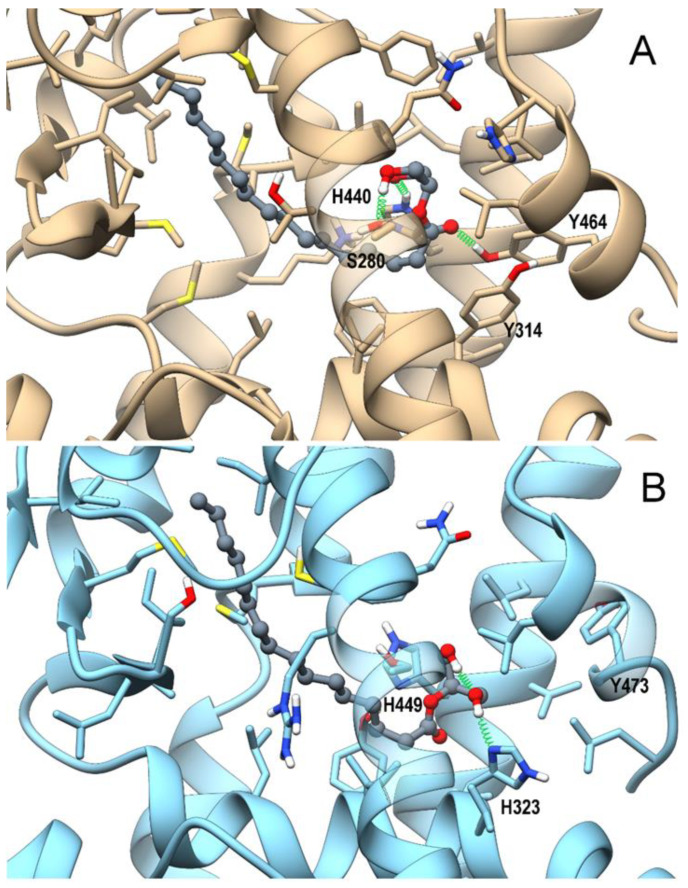
Theoretical complexes of PPARα (tan) (**A**) and PPARγ (light blue) (**B**) with 2-PG colored in dark grey and shown in ball & stick representation. Protein residues within 5 Å from the ligands are shown in stick representation. H-bonds are shown as green springs. Hydrogen, nitrogen, oxygen, and sulfur atom are painted white, blue, red, and yellow, respectively. A transparent surface for ribbons was used wherever they hide the ligand-binding site.

The combination of the pivotal role played by PPARs in inflammation, lipidic and glycemic metabolism, and previously-observed interactions of eCBome-related congeners with PPARα and PPARγ [26,63] prompted us to perform an in silico study to evaluate whether 2-PG could act as a ligand at these nuclear receptors, using a combined approach of molecular docking and molecular dynamic (MD) simulations. As shown in Figure 4A,B, 2-PG in PPARα can recapitulate the polar interactions of a canonical agonist, involving a network of H-bonds with His440 (helix H10/11), Ser208 (Helix H3), and especially with Tyr464 (helix H12), a residue critical for PPAR full activation.

**Figure 5:**
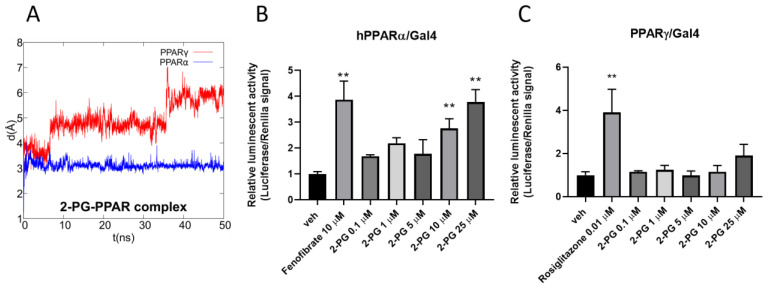
(**A**) Root-mean-square deviation (RMSD) plot of 2-PG in complex with PPARα/γ over the 50 ns of molecular dynamics (MD) trajectory after best fitting of protein backbones. Luciferase assays performed in PPARα- or PPARγ-transfected COS cells (**B**,**C**). Bar graphs showing the ratio between firefly and *Renilla* luciferase in response to increasing concentrations of 2-PG. Fenofibrate and rosiglitazone were used as positive controls for PPARα (**B**) and PPARγ (**C**), respectively. The vehicle group was set to 1; thus, the relative luciferase activities obtained for each tested compound and concentration are presented as fold induction in comparison to the vehicle control. Each point is the mean ± SEM of four separate determinations performed in duplicate. The double asterisk denotes a *p*-value ≤ 0.005 versus the vehicle group.

The alky chain is hosted in the hydrophobic pocket formed by the helix bundle and the β-sheet and wraps around helix H3. Indeed, the interaction with Tyr464 stabilizes the conformation of helix H12, and is thus a “marker” for full agonist, being propaedeutic to the co-activator recruitment. Instead, the only stable H-bond occurring in PPARγ during MD involves His323 (helix H5, corresponding to PPARα Tyr314), whereas Tyr473 (corresponding to PPARα Tyr464) on helix H12 is pushed away. This weaker interaction pattern translates into a higher mobility of 2-PG within the PPARγ binding site during MD in comparison with that observed in the PPARα complex, (Figure 5A).

Taken together, these data strongly suggest a higher activity/selectivity of 2-PG toward PPARα in comparison with PPARγ. Then, to validate the in silico data, luciferase assay experiments were carried out on both PPAR isoforms. The results showed that 2-PG increased luciferase activity in a concentration-dependent manner only in PPARα transfected cells (Figure 5A). Notably, the extent of luciferase activity observed with 25 µM of 2-PG was comparable to that of fenofibrate (10µM) a potent canonical agonist of PPARα (Figure 5B). On the contrary, no significant effects of 2-PG were observed in cells transfected with PPARγ (Figure 5C). This indicates that the compound is an agonist for PPARα, but not for PPARγ, as predicted by the in silico analysis. Of note, 1-PG could not be tested in vitro because it was not commercially available.

### 3.5. In Silico Identification 1-PG as a Potential Endogenous Ligand of PPARα/γ

**Figure 6:**
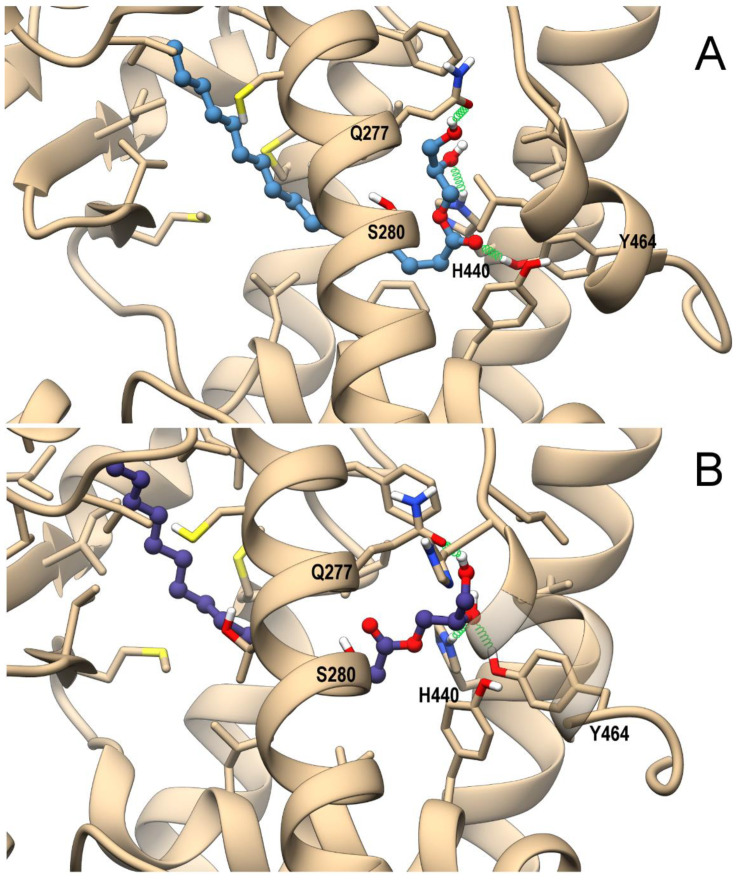
Theoretical complexes of PPARα (tan) with D-PG (steel blue) (**A**) and L-PG (slate blue) (**B**) with 1-PG shown in ball & stick representation. Protein residues within 5 Å from the ligands are shown in stick representation. H-bonds are shown as green springs. Hydrogen, nitrogen, oxygen, and sulfur atom are painted white, blue, red, and yellow, respectively. A transparent surface for ribbons was used wherever they hide the ligand-binding site.

The potential ability of 1-PG to bind and activate both PPAR isoforms was evaluated by the same computational approach used for 2-PG. The representative MD frames of the complexes of PPARα and PPARγ with either the *D*- or *L*- enantiomers of 1-PG are shown in Figure 6A,B and Supplementary Figure S5, respectively.

As for 2-PG, both 1-PG enantiomers engage in PPARα H-bonds with H440 (H10/11), Y464(H12), and S280(H3) within the ligand-binding domain (LBD), albeit the H-bond with Y464(H12) has a lower occurrence in comparison with 2-PG (<30% vs. ~80%, respectively), due to the carbonyl group flipping between Y464(H12) and Ser280(H3) for D-1-PG and the alternate involvement of the same hydroxy group in H-bond with either Y464(H12) or H440(H10/11) for L-1-PG. The reduced stability of such H-bond in both PPARα complexes could in principle affect the potency of 1-PG toward this receptor in comparison with 2-PG (Figure 6 A,B). As for the PPARγ complex, 1-PG forms stable H-bonds with Y473(H12), differently from 2-PG. In particular, D-1-PG engages H-bonds with both H440(H10/11) and Y473(H12) (Supplementary Figure S5A), even though its alkyl chain does not adopt the typical horseshoe conformation around H3 observed in canonical agonists, pointing instead toward helix 5. The optimal orientation of the alkyl chain is observed in L-1-PG, which, however, does not optimize its polar interaction, being involved in a single intermolecular H-bond with Y473(H12) (Supplementary Figure S5B). Thus, the two enantiomers show different arrangements within the LBD that in principle could reflect a different ability in triggering receptor activation. Of note, 1-PG could not be tested in vitro because it was not commercially available in an enantiomerically pure form.

In summary, these data suggest that both enantiomers of 1-PG could be endowed with agonist activity at PPARα.

## 4. Discussion

*A. muciniphila* intrigued and inspired researchers from the very beginning of its discovery. While the research in pre-clinical models continues to increase the amount of hard evidence in favor of the health benefits of the bacterium (reviewed in [2,6,7,8,9]), our team decided to take the plunge into human clinical investigation and conducted the first controlled pilot study consisting in the daily administration of either alive or pasteurized form of the bacterium. Our first human data validated the feasibility and the safety of supplementation in overweight and obese individuals newly diagnosed with prediabetes and metabolic syndrome [20]. While this first study highlighted positive metabolic effects, its primary objective was not focused on identifying underlying mechanisms behind these effects. The present study was therefore conceived with the idea of providing new mechanistic answers, toward the identification of novel cross-talks between the bacterium and biological systems, and the eCBome in particular. As a reminder, the eCB system consists of cannabinoid receptors, their endogenous lipid ligands, and ligand-metabolizing enzymes, while the enlarged eCB system, referred to as the eCBome, includes the eCB system plus a plethora of additional eCB-like mediators, together with their receptors, metabolizing, and catabolic enzymes [26]. The ability of this latter complex system to influence central and peripheral functions such as appetite, feeding behavior, and energy balance has recently received considerable attention, particularly in the light of metabolic disorders such as metabolic syndrome [23,25,68,69]. Since these same metabolic functions are under the influence of the intestinal microbiota, the scientific community has been paying increasing interest in the potential cross-talk between the eCBome, the gut microbiome, particularly during gut dysbiosis in the context of obesity [28,33,40].

The first objective of this study was to investigate potential eCBome-related changes induced by 3 months of daily *A. muciniphila* supplementation in overweight and obese individuals. For this purpose, we used both multivariate and univariate approaches, as previous research has shown the added value of combining both approaches to unravel new scientific questions [70]. The plasmatic concentrations of 25 endogenous eCBome-related lipids were measured at baseline and the end of the intervention using LC-MS/MS. Quantification of the eCBome profile was then coupled with multivariate statistical analyses including PCA and PLS-DA. Baseline characterization of the quantified eCBome by PCA showed that there was no significant difference between groups at baseline. When PCA and PLS-DA were performed on the post-treatment eCBome, both methods revealed groups overlapping in the two-dimension plane summarizing the highest variance in the quantified eCBome. Lack of separation between groups at 3 months, and between timings within each group aligned to indicate that the intervention did not induce an overall switch of the eCBome profile, regardless of the administered form of *A. muciniphila*. The invalidity of the PLS-DA model did not allow the identification of relevant eCB congeners that would have discriminated among the groups. Of note, participants were treatment-naïve, meaning that they were not treated for their clinical condition during the 3 months period. Despite this and even though metabolic syndrome is a progressive disease; no changes were measured in the eCBome profile in the placebo group. We next investigated whether the *A. muciniphila* relative abundance was associated with eCB-like mediators for both timings, and found no significant correlations. This result somehow echoes the outcomes of a previous study in which several bacteria were correlated to eCBome-related mediators in a large cohort of individuals with varying degrees of body mass index and adiposity and no intervention [33]. In that study, the relative abundance of the *Akkermansiaceae* was poorly correlated with most of the quantified eCB congeners. However, the authors found a significant correlation with 2-EPG [33]. Of note, our results were generated in a fasting condition. As it is known that circulating eCBome mediators are influenced by the nutritional status and nutrient intake, we do not exclude that a non-fasting state could have yielded similar results had all participants ingested the exact same meal in terms of quantity and composition.

Although the overview given by the heat map did not seem to indicate the presence of specific or major changes, we wanted to dwell more deeply in the statistical analyses by measuring the average fold changes for each of the lipid mediators, within each group. The results prompted us to investigate further some of the eCB-like mediators, and subsequent univariate analyses showed that treatment with the alive *A. muciniphila* significantly modulated the blood levels of 1-PG and 2-PG in comparison to the placebo group. More specifically, the pasteurized form significantly prevented the reduction of 1-PG observed in the placebo group without inducing a clear increase in the levels of those compounds. Of note, this divergence of effects between the two administrated forms of the bacterium with regard to the eCBome aligned with previous reports suggesting that the pasteurized form does not influence the metabolism via the same metabolic pathways as the alive form [11,12,20]. Although clear mechanisms behind these differential impacts are not clearly delineated to this date, we do not exclude that they are the consequence of differential metabolizing activities between the two forms.

MAGs usually exist as three isoforms in animal tissues: the 2-MAGs, in which the fatty acid is esterified to the 2-position of glycerol, and the 1-MAGs, in which the fatty acid (which is very seldom polyunsaturated) is instead esterified to the 1-position. However, 2-MAGs can spontaneously isomerize to 1- and 3-MAGs due to fatty acid migration from the 2- to either the 1- or 3-position of glycerol [71], which may occur in vivo or during the extraction and purification process. 1- and 3-MAGs are enantiomers, and our analytical method does not allow us to distinguish between the two enantiomers, both of which can be artifactually derived from the corresponding 2-MAGs anyway. Thus, 1-MAGs extracted are usually enantiomeric mixtures. Using in silico analysis, we discovered that both enantiomers (D and L) of 1-PG are potential activators of PPARα and, likely less efficaciously, PPARγ, whereas the same analysis predicted for 2-PG selective agonist activity for PPARα.

To explore the molecular, and therefore physiological implications that *A. muciniphila*-induced relative elevation of 1- and 2-PG circulating levels might have in the context of metabolic syndrome, and to validate in vitro the predictions of the in silico approach used, we next investigated the effect of 2-PG on PPARα and PPARγ functional assays. Both nuclear receptors are considered as targets for other eCBome mediators, particularly for AEA and/or some of its congeners such as PEA and OEA [72], which can thereby act as key regulators of lipid metabolism, among other physiological processes [73]. For instance, previous work proposed that activation of PPARα by NAE might mediate the anorexigenic, lipolytic, and anti-inflammatory effects of these compounds [48,74,75,76,77]. AEA instead was found to activate PPARγ although at concentrations higher than those needed to activate cannabinoid receptors [50]. However, no equivalent data existed so far regarding MAGs, such as 1- and 2-PG. Our luciferase assay data identify 2-PG as an agonist for PPARα activation as effective as fenofibrate, and selective over PPARγ. Of note, 2-PG was inactive up to 25 µM on the human recombinant TRPV1 channel overexpressed in HEK-293 cells (data not shown), another reported target for 2-MAGs [78]. Nevertheless, our data regarding PPAR activation perfectly validate what was expected from the in silico analysis, and, therefore, also indirectly suggest that 1-PG, for which we could not find an enantiomerically pure commercial source to be tested in vitro, is also a likely PPARα agonist, for which, however, a possibly weaker activity at PPARγ could also be predicted (see also [63] as another example of how our in silico approach is usually perfectly validated by in vitro functional luciferase assays). These two compounds together were found here to have, in our cohort, circulating concentrations in the µM range, which is compatible with the concentrations needed for them to activate PPARα. These results emphasize the potential metabolic importance of the modulation of the plasma levels of 1- and 2-PG induced by *A. muciniphila*.

Indeed, as mentioned above, we have previously shown that oral administration of *A. muciniphila* in obese mice increased intestinal endogenous production of 2-PG [10]. Our present data, thus, extend for the first time this finding to human overweight and obese individuals. Although we cannot assume yet a direct 1/2-PG mediated mechanism, the supplementation also resulted in the restoration of PPARα expression in the adipose tissue [10]. In line with this, other in vivo studies have shown an increased adipocyte or hepatic expression of PPARα in other experimental set-ups eliciting direct or indirect *A. muciniphila* increased abundance in the intestine [79,80,81]. Thus, we postulate that *A. muciniphila* might exert part of its beneficial effects in a PPARα-dependent manner, through increased endogenous levels of the agonist 2-PG, and potentially 1-PG. Although our formulated hypothesis is consistent with the aforementioned literature, it is important to emphasize that these observations might have resulted also from the modulation of other several mediators, belonging either to the eCBome and not measured here (such as *N*-acyl-amino acids, primary amides, and *N-*acyl-neurotransmitters, which were mostly below the detection limit in our LC-MS/MS analyses) or to other signaling systems. It is nevertheless interesting to note that a certain number of physiological effects observed during our first study, yet less pronounced, are still analogous to the expected effects induced by endogenous/exogenous activation of PPARα in the context of obesity (i.e., reduced dyslipidemia, lesser inflammation, and decreased insulin resistance) [20,43]. At any rate, our data do not conclusively demonstrate activation of PPARα by *A. muciniphila*, thus, warranting further studies to confirm eCBome-mediated activation of PPARα in the presence of *A. muciniphila*.

In conclusion, this extended mechanistic study allowed us to demonstrate that *A. muciniphila* exerts its beneficial effects on metabolism independently from generalized changes in the plasmatic eCBome mediators in the context of the metabolic syndrome. However, oral supplementation with the alive bacterium significantly prevented the reduction of 2-PG and 1-PG levels observed upon the progression of the disease. Deeper in silico and in vitro investigations lead to the identification of PPARα as a molecular target for 2-PG (and probably 1-PG), through which part of *A. muciniphila*’s metabolic effects might have been mediated. This potential new underlying mechanism requires to be further investigated in future studies but constitutes a good candidate to explain part of the metabolic effects induced by the alive bacterium.

## Figures and Tables

**Table 1:** Endocannabinoids and endocannabinoids-related congener’s abbreviations.

Endocannabinoids and Related Mediators	
1-PG	2-palmitoyl-glycerol
2-PG	1-palmitoyl-glycerol
1-PG & 2-PG	1-palmitoyl-glycerol + 2-palmitoyl-glycerol
2-OG	1(3)- and 2-oleoylglycerol
2-LG	1(3)- and 2-linoleoyl-glycerol
2-AG	1(3)- and 2-arachidonoyl-glycerol
2-DHG	1(3)- and 2-docosahexaenoyl-glycerol
2-EPG	1(3)- and 2- eicosapentaenoyl-glycerol
2-DPG	1(3)- and 2-docospentaenoyl-glycerol(n − 3)
PEA	*N*-palmitoyl-ethanolamine
SEA	*N*-stearoyl-ethanolamine
OEA	*N*-oleoyl-ethanolamine
LEA	*N*-linoleoyl-ethanolamine
AEA	*N*-arachidonoyl-ethanolamine
EPEA	*N*-eicosapentaenoyl-ethanolamine
DPEA	*N*-docosapentaenoyl-ethanolamine(n − 3)
DHEA	*N*-docosahexanoyl-ethanolamine
AA	arachidonic acid
EPA	eicosapentaenoic acid
DPA	docosapentaenoic acid (n − 3)
DHA	docosahexaenoic acid

“n − 3” correspond to omega 3 fatty acid.

**Table 2:** PLS-DA cross-validation details for the first 5 components. PLS-DA were performed to cluster groups according to the eCBome quantified at the end of the intervention. Reported Accuracy, R2, and Q2 according to the first 5 components. The analyses were performed using MetaboAnalyst v4.0 (accessed date: 26 November 2020).

Measure	1 Comps	2 Comps	3 Comps	4 Comps	5 Comps
Accuracy	0.374	0.313	0.344	0.344	0.313
R2 ^a^	0.14165	0.339	0.382	0.410	0.427
Q2 ^b^	−0.1494	−0.852	−1.015	−1.309	−1.985

^a^ The calculated R2 is the multiple correlation coefficient. ^b^ The calculated Q2 is an estimate of the predictive ability of the model. Abbreviations: Comps, components.

## Data Availability

The data presented in this study are available on request from the corresponding authors.

## Supplementary Materials

The following are available online at https://www.mdpi.com/2073-4409/10/1/185/s1, Figure S1: Time series principal component analysis representing the 3 main components, Figure S2: Focalized principal component analysis focused on *A. muciniphila* abundance, Figure S3: Pairwise PCA and PLS-DA score plots, Figure S4: Two-dimensional PCA and PLS-DA score plots, Figure S5: Theoretical complexes of PPARγ with D-PG and L-PG.
