# Supplementary material for: Beneficial Effects of Akkermansia muciniphila Are Not Associated with Major Changes in the Circulating Endocannabinoidome but Linked to Higher Mono-Palmitoyl-Glycerol Levels as New PPARα Agonists
Source: Cells. 2021 Jan 19;10(1):185. doi: 10.3390/cells10010185 (PMC7832901; doi:10.3390/cells10010185)
Supplement: Supplementary file 1 [file cells-10-00185-s001.pdf]

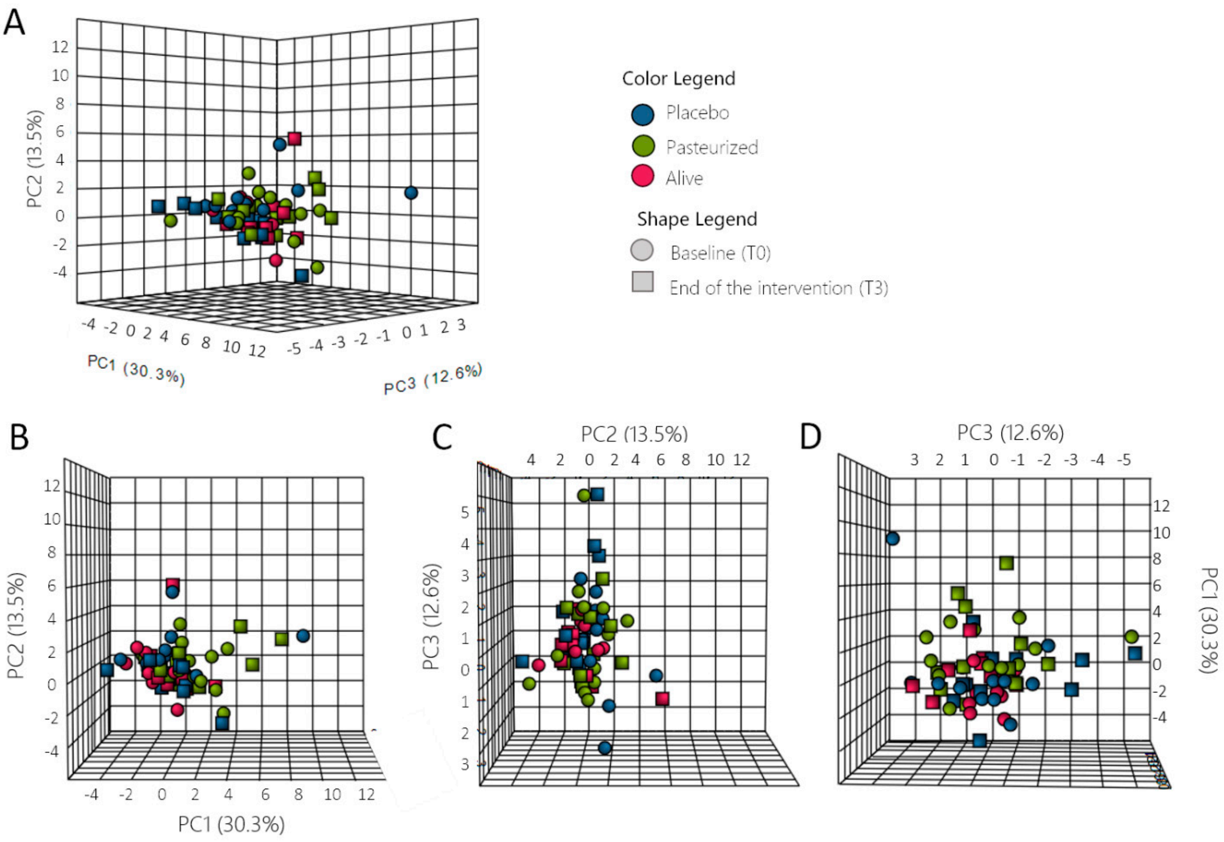

Supplementary Figure S1. Time series principal component analysis representing the 3 main components. Each participant is represented twice, with respect to the two time points of the study. (**A**) Global visualization of the sample plot according to the 3 dimensions. (**B**) Dimension 1 versus dimension 2. (**C**) Dimension 2 versus dimension 3. (**D**) Dimension 1 versus dimension 3. Placebo group, $n = 11$; pasteurized bacteria group, $n = 12$; alive bacteria group, $n = 9$.

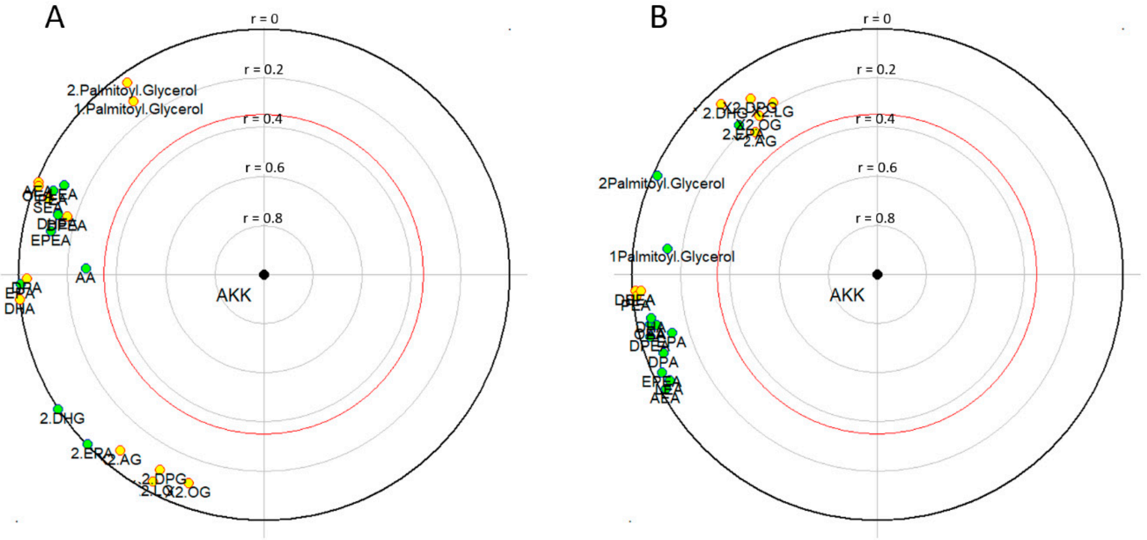

Supplementary Figure S2. Focalized principal component analysis focused on *A. muciniphila* abundance. The graphs show correlation between *A. muciniphila* abundance and eCBs-related mediators at baseline (**A**) and at the end of the intervention (**B**). Yellow dots indicate eCB-related mediators negatively correlated to *A.muciniphila* abundance; green dots correspond to eCB-related mediators positively correlated to *A.muciniphila* abundance. The correlation is significant only if the dot is inside the red circle, corresponding to the cutoff point of a p value of 0.05. The concentric circles represent the R^2 of the

correlation. The fpca also graphically display the correlation existing between the eCBs, variables close to each other being highly correlated, while variable in opposite direction with regard to the center being negatively correlated. Abbreviations: AA, arachidonic acid; AKK, *A. muciniphila*; AEA, N-arachidonoyl-ethanolamine; 2.AG, 1(3)- and 2-arachidonoyl-glycerol; DHA, docosahexaenoic acid; DHEA, N-docosahexanoyl-ethanolamine; 2.DHG, 1(3)- and 2-docosahexaenoyl-glycerol; DPA, docosapentaenoic acid (n–3); DPEA, N-docosapentaenoyl-ethanolamine(n–3); 2.DPG, 1(3)- and 2-docospentaenoyl-glycerol(n − 3); 2.EPA, eicosapentaenoic acid; EPEA, N-eicosapentaenoyl-ethanolamine; 2.EPG, 1(3)- and 2- eicosapentaenoyl-glycerol; LEA, N-linoleoyl-ethanolamine; 2.LG, 1(3)- and 2-linoleoyl-glycerol; OEA, N-oleoyl-ethanolamine 2.OG, 1(3)- and 2-oleoylglycerol; PEA, N-palmitoyl-ethanolamine; SEA, N-stearoyl-ethanolamine.

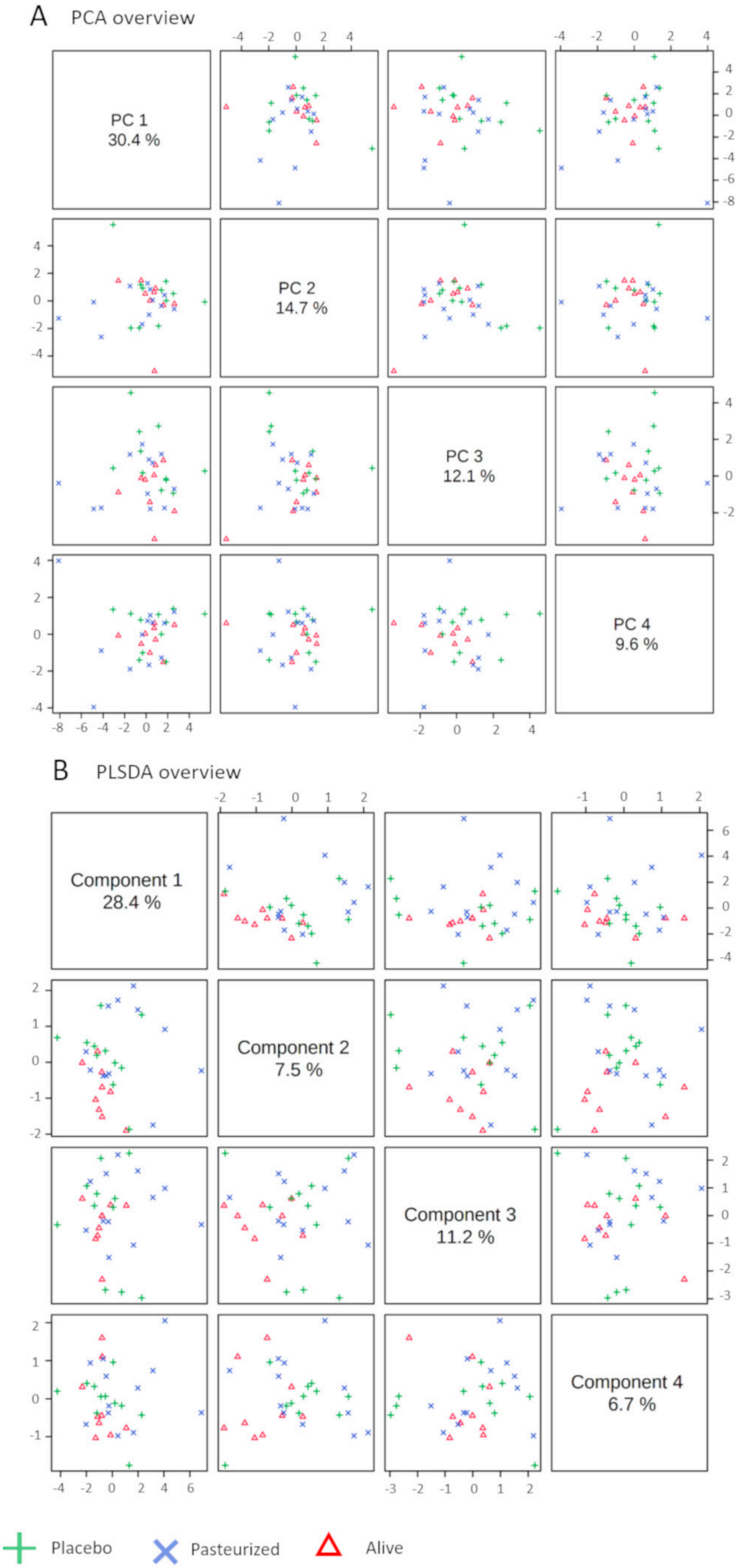
A PCA overview
PC 1
30.4 %
PC 2
14.7 %
PC 3
12.1 %
PC 4
9.6 %
B PLSDA overview
Component 1
28.4 %
Component 2
7.5 %
Component 3
11.2 %
Component 4
6.7 %
Placebo
Pasteurized
Alive

Supplementary Figure S3. Pairwise PCA (**A**) and PLS-DA score plots (**B**) of the time 3 months quantified eCBome, for the first 4 principal components/dimension. Color code: green dots: individuals from the placebo group; blue dots: individuals from the pasteurized group; red dots: individuals from the alive group. The graphs were generated by the PCA and PLSDA module of MetaboAnalyst 4.0 web portal. Placebo group, $n = 11$; pasteurized bacteria group, $n = 12$; alive bacteria group, $n = 9$.

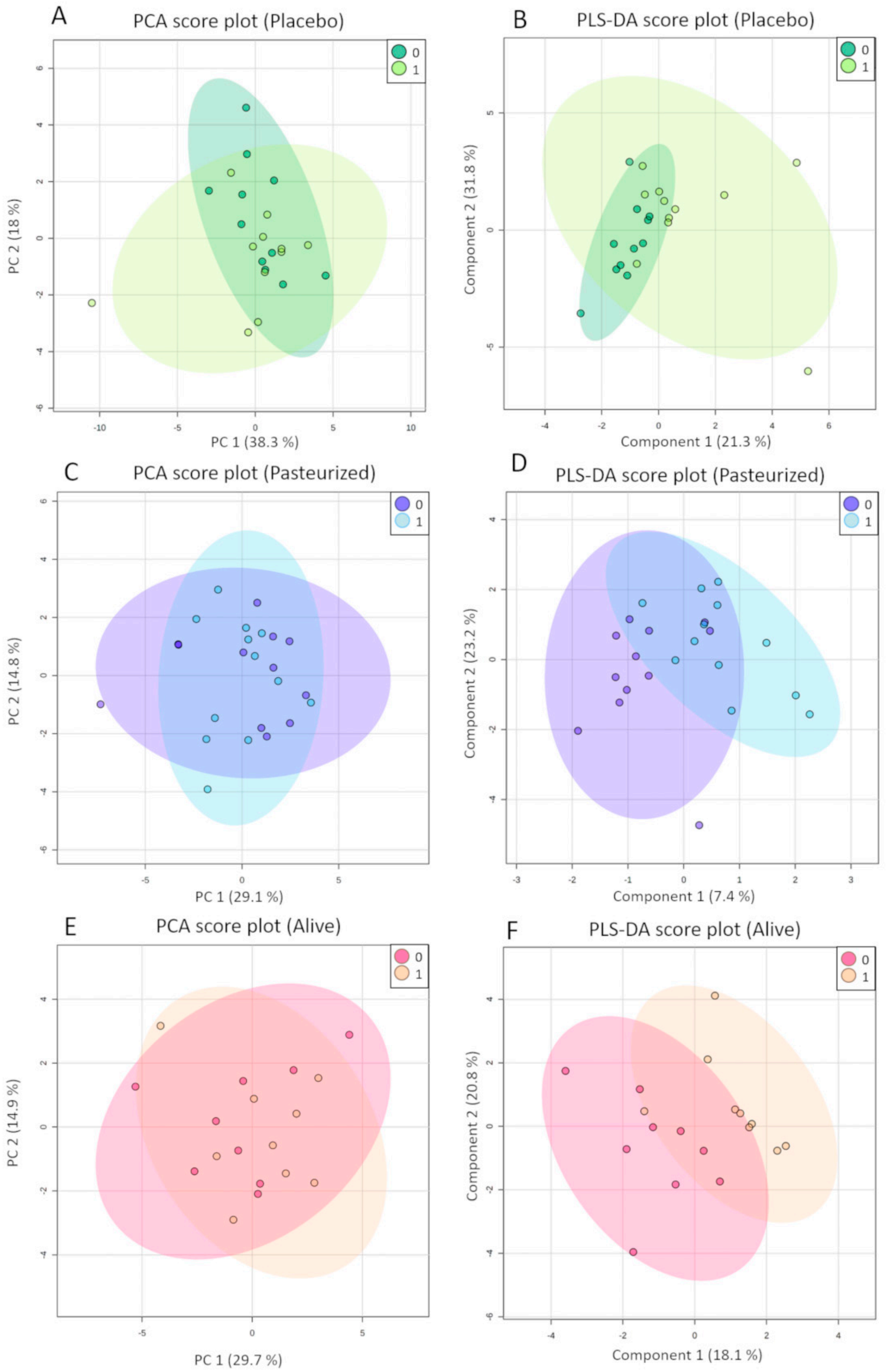

Supplementary Figure S4. Two-dimensional PCA (**A**,**C**,**E**) and PLS-DA (**B**,**D**,**F**) score plots obtained on the basis of the ECB profile quantified at baseline and following the treatment, according to groups. (**A**) PCA Score plots for the placebo group. (**B**) PLS-DA score plots for the placebo group, T0 versus T3. (**C**) PCA Score plots for the Pasteurized group, T0 versus T3. (**D**) PLS-DA

Score plots for the Pasteurized group, T0 versus T3. (E) PCA score plot for the alive group, T0 versus T3. (F) PLS-DA score plot for the alive group, T0 versus T3. The semi-transparent areas are the 95% confidence regions for each timing. Color code: lights ellipses correspond to baseline eCBome profile and darker ellipse correspond to time 3 months eCBome profile. The graphs were generated by the PCA and PLSDA module of MetaboAnalyst 4.0 web portal. Placebo group, *n* = 11; pasteurized bacteria group, *n* = 12; alive bacteria group, *n* = 9.

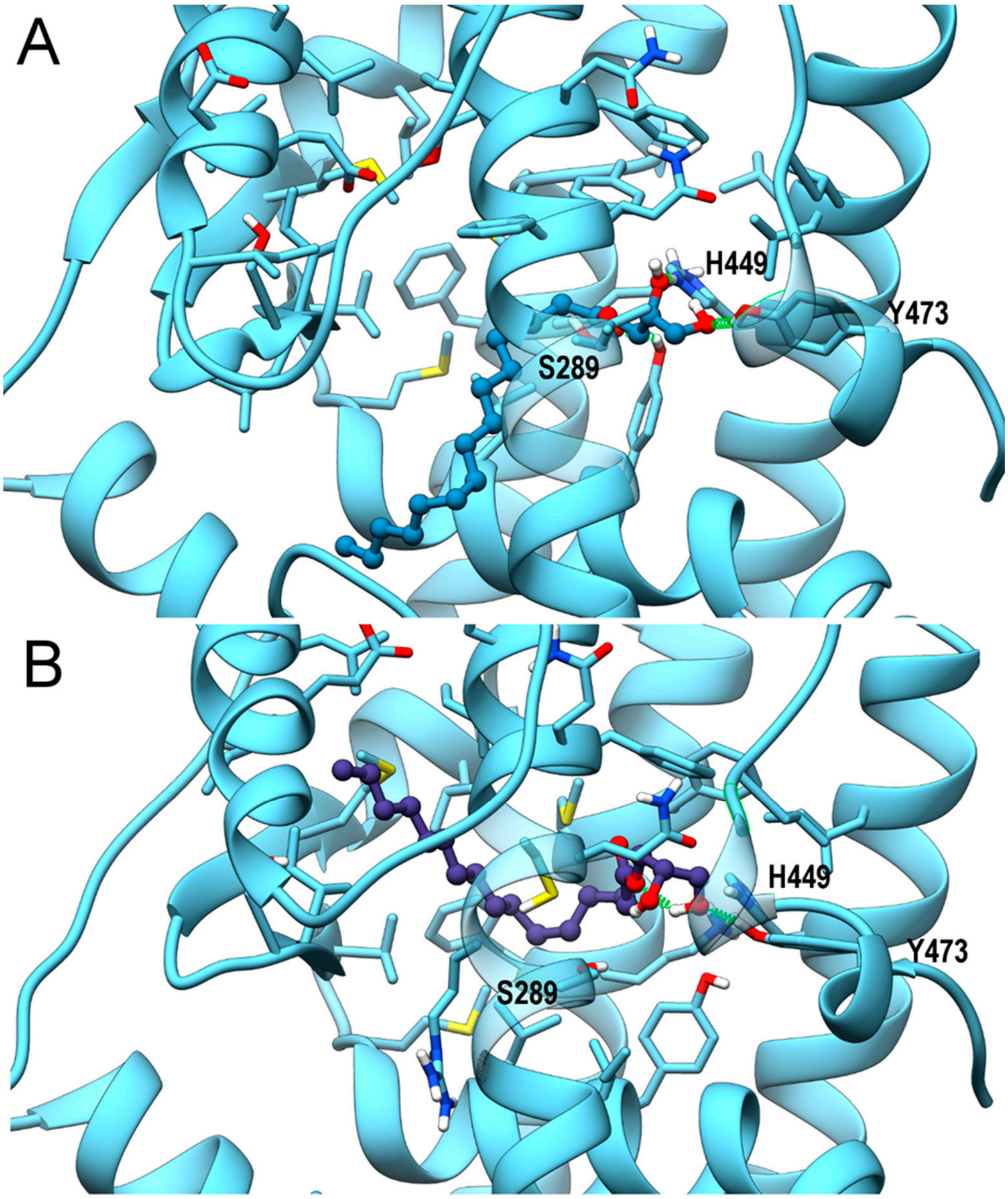

Supplementary Figure S5. Theoretical complexes of PPARγ (light blue) with D-PG (steel blue) (**A**) and L-PG (slate blue) (**B**) with 1-PG shown in ball&stick representation. Protein residues within 5 Å from the ligands are shown in stick representation. H-bonds are shown as green springs. Hydrogen, nitrogen, oxygen and sulphur atom are painted white, blue, red and yellow, respectively. Transparent surface for ribbons was used wherever they hide the ligand binding site.
